# *ARID1A* Variations in Cholangiocarcinoma: Clinical Significances and Molecular Mechanisms

**DOI:** 10.3389/fonc.2021.693295

**Published:** 2021-06-25

**Authors:** Shankun Zhao, Youwen Xu, Weizhou Wu, Pan Wang, Yichao Wang, Hao Jiang, Jie Zhu

**Affiliations:** ^1^ Department of Urology, Taizhou Central Hospital (Taizhou University Hospital), Taizhou, China; ^2^ Department of Clinical Laboratory, Taizhou Central Hospital (Taizhou University Hospital), Taizhou, China; ^3^ Department of Urology, Maoming People’s Hospital, Maoming, China; ^4^ Department of Hepatobiliary Surgery, Taizhou Central Hospital (Taizhou University Hospital), Taizhou, China

**Keywords:** *ARID1A* variations, cholangiocarcinoma, biomarker, prognosis (carcinoma), pathogenesis

## Abstract

Cholangiocarcinoma (CCA), a high mortality malignant carcinoma characterized by advanced disease and frequent recurrence, constitutes a major challenge for treatment and prognosis. AT-rich interaction domain 1A (*ARID1A*) variation is a distinct genetic entity in CCA, getting mounting concerns recently. Here, we comprehensively reviewed the clinical significance and molecular mechanisms of *ARID1A* alterations in CCA. Based on the independent data derived from 29 relevant studies, the variation rate of *ARID1A* in intrahepatic and extrahepatic CCA is reported at 6.9–68.2% and 5–55%, respectively. Most of the included studies (28/29, 96.6%) suggest that *ARID1A* serves as a tumor suppressor in CCA. *ARID1A* variation may be an important prognostic indicator to predict disease mortality, metastasis, and recurrence in patients with CCA. Multifactorial molecular mechanisms are involved in the relationship between *ARID1A* variations and the pathogenesis and pathophysiology of CCA, including disruption of the cell cycle, chromatin remodeling, oxidative stress damage, DNA hypermethylation, and the interaction of multiple genes being affected. This review describes that *ARID1A* variation might be a potential diagnostic and prognostic biomarker for CCA. Future diagnoses and treatments targeting *ARID1A* hint towards a precision medicine strategy in the management of CCA.

## Introduction

Cholangiocarcinoma (CCA) is the second most frequently diagnosed primary liver malignancy, accounting for 10–20% of primary hepatic carcinomas, and representing 3% of all gastrointestinal tumors (1). Accounting to the anatomic location of biliary tree involvement, CCA can be classified into intrahepatic (ICC, 6–8% of CCA) and extrahepatic (ECC, including perihilar and distal CCA) (2). CCA is a highly malignant neoplasm resulting from the malignant transformation of the epithelium of the biliary tract, of which approximately 90% are adenocarcinoma (3). The incidence and mortality rates of CCA has steadily increased in recent decades. The rising incidence is considered to be related to the burden of hepatitis B and hepatitis C virus infection (4). In addition, primary sclerosing cholangitis, obesity, diabetes, inflammatory bowel disease, non-alcoholic fatty liver diseases, and liver fluke infestation are also the key risk factors for CCA development (5). As reported, only 10–15% of CCA patients are eligible for curative surgery (6). Due to the high rates of disease recurrence (50–60%), the 5-year overall survival rates are only 30% even after surgery (7). Furthermore, locally advanced and distant metastasis terrifically contribute to the high mortality of CCA patients. Therefore, it is necessary to look for potential biomarkers that could help with identifying the disease prognosis.

Heterogeneity in cancer aggressiveness and prognosis of CCA may be driven by the differential alteration of the genetic variations. It is reported that genes encoding components of the SWItch/Sucrose Non-Fermenting (*SWI/SNF*) chromatin-remodeling complex may be one of the most commonly mutated genes in multiple malignancies. *SWI/SNF* chromatin remodeling complex is involved in transcription and DNA replication and repair (8). The AT-rich interaction domain 1A (*ARID1A*) is the most frequently mutated *SWI/SNF* gene across a broad spectrum of human cancers, which facilitates access of proteins to DNA (9, 10). *ARID1A* is presumed to be a tumor suppressor based on loss-of-function mutational profiles observed in many cancers (11), including gastrointestinal cancers. It was reported that *ARID1A* variations were present in 18.7% of gastric, 13.7% of hepatocellular, 9.4% of colorectal, and 3.6% of pancreatic cancers (12). Most of the *ARID1A* variations are inactivating alterations, leading to loss of *ARID1A* protein expression (13). In recent years, accumulated evidence indicates that *ARID1A* variation is associated with clinicopathologic features of CCA (14, 15). *ARID1A* variations were identified in 7.2 to 36% of ICC and 5 to 12.3% of extrahepatic CCA (16).

At present, the exact role of *ARID1A* on the prognosis and clinicopathologic features of CCA is still controversial among different clinical studies. It was suggested that *ARID1A* had dual roles in both oncogenicity and tumor suppression in CCA. Based on the published data, most studies indicated *ARID1A* may be a tumor suppressor gene. For example, Yang et al. (17) demonstrated that low expression of *ARID1A* was associated with worse prognosis in intrahepatic CCA than those with high expression. However, a recent study developed by Bi et al. (18) indicated that high expression of *ARID1A* might be correlated with worse prognosis in intrahepatic CCA patients than those with low expression. The effect of *ARID1A* in CCA is currently inconclusive. In this review, we aim to summarize all the evidence on the association between *ARID1A* variations or expression and CCA development, as well as open up currently known mechanisms therefore to facilitate clinical understanding of the role of *ARID1A* in CCA.

## Alteration Of *ARID1A* In CCA Among Different Relevant Studies

Four databases, i.e., MEDLINE (PubMed), EMBASE (OVID), Cochrane Library, and PsychINFO were systematically searched to screening the related studies prior to April 1, 2021. Only studies reporting with English language were included. The searching strategy employed for identifying the eligible studies in PubMed databases was: ((((((((((((((“Cholangiocarcinoma”[Mesh]) OR (Cholangiocarcinomas)) OR (Cholangiocellular Carcinoma)) OR (Carcinoma, Cholangiocellular)) OR (Carcinomas, Cholangiocellular)) OR (Cholangiocellular Carcinomas)) OR (Extrahepatic Cholangiocarcinoma)) OR (Cholangiocarcinoma, Extrahepatic)) OR (Cholangiocarcinomas, Extrahepatic)) OR (Extrahepatic Cholangiocarcinomas)) OR (Intrahepatic Cholangiocarcinoma)) OR (Cholangiocarcinoma, Intrahepatic)) OR (Cholangiocarcinomas, Intrahepatic)) OR (Intrahepatic Cholangiocarcinomas)) AND ((((((((((((((*ARID1A*) OR (B120)) OR (BAF250)) OR (BAF250a)) OR (BM029)) OR (C1orf4)) OR (CSS2)) OR (ELD)) OR (MRD14)) OR (OSA1)) OR (P270)) OR (SMARCF1)) OR (hELD)) OR (hOSA1)). Additional studies were detected by manual inspection of reference lists in the relevant publications. The following information was extracted based on a data collection form, including the first authors’ names of the included studies, publication year, country, type of cholangiocarcinoma, *ARID1A* variations presented with cases and percentage, effect of *ARID1A* on CCA, and clinical implications or biological functioning of *ARID1A*.

**Figure 1:**
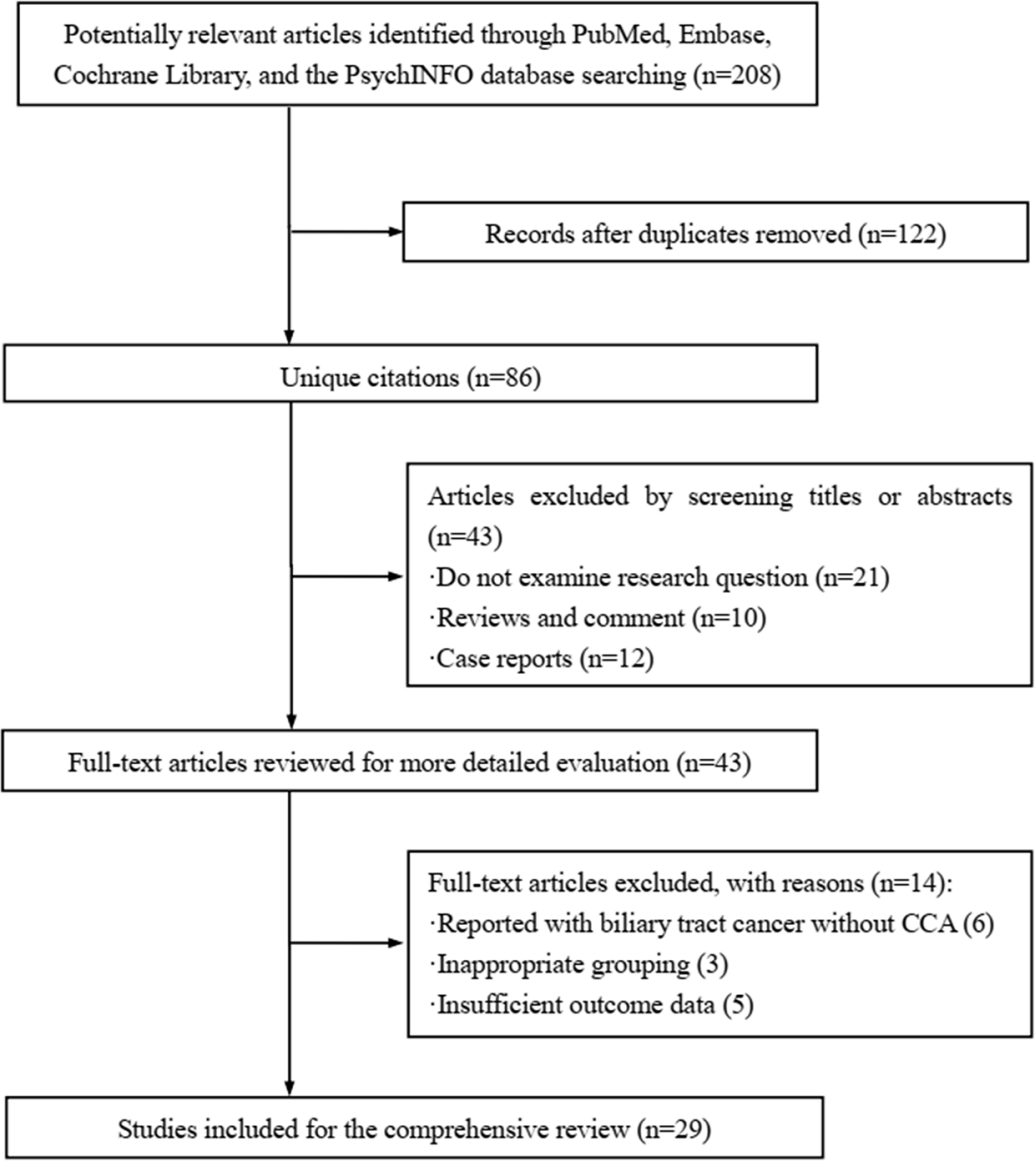
Flowchart of study selection.

**Figure 2:**
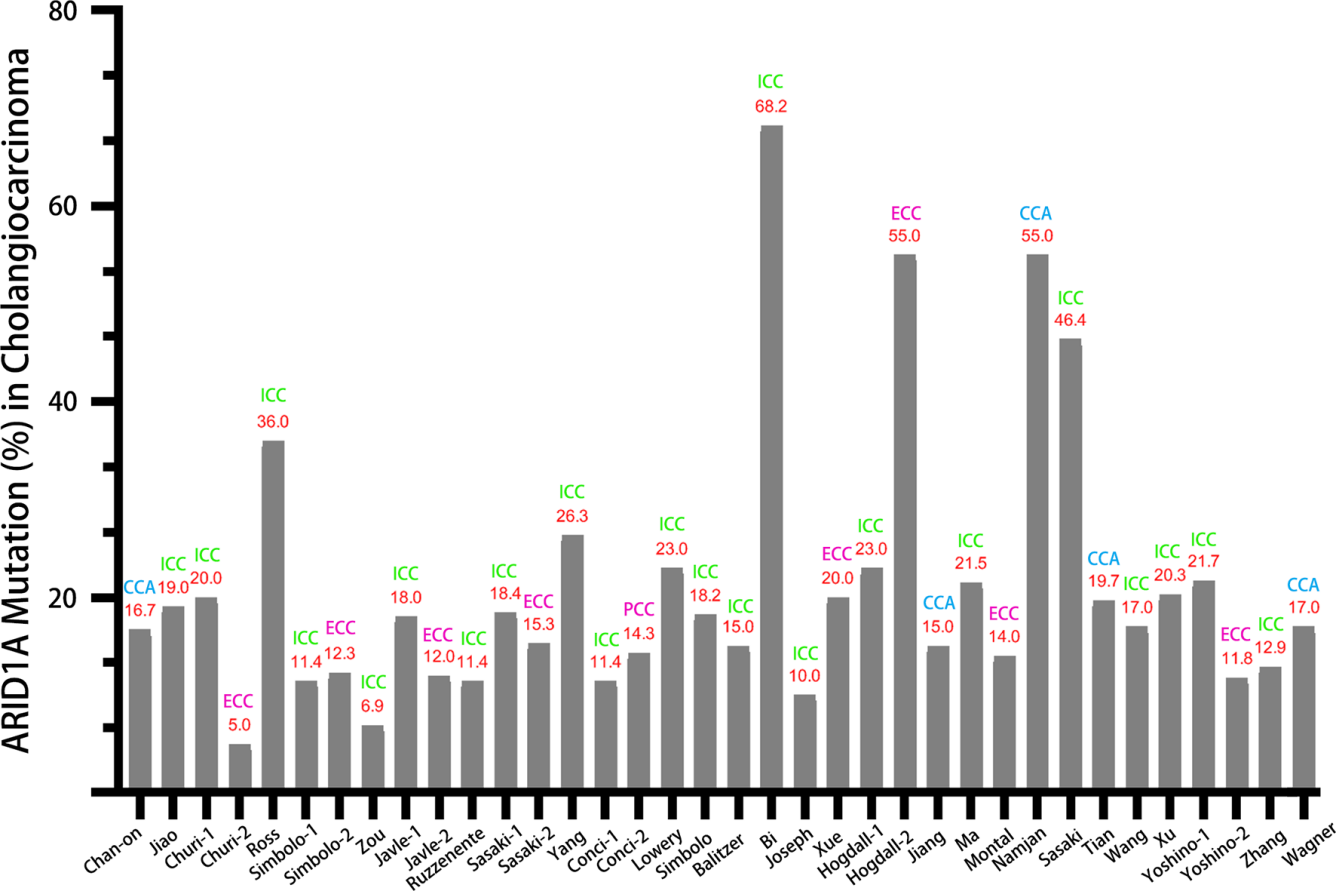
Th*e* variation rate of *ARID1A* in CCA, ICC, and ECC.

**Figure 1** displayed the search flowchart for identifying the eligible studies reporting *ARID1A* variants and CCA. In the initial database search, 208 publications were detected, of which 82 from MEDLINE, 47 from EMBASE, 41 from the Cochrane Library, and 38 from the PsychINFO database. Finally, 29 eligible studies (16–44) with a total of 2,945 subjects were included. The publication years of the included studies ranged from 2013 to 2021. Seven studies were conducted in Europe, 15 studies in Asia, and 7 studies in America. The cancer type in those eligible studies included CCA (reported in 5 studies), ICC (reported in 22 studies), and ECC (reported in 9 studies). The sample size ranged from 7 to 412 patients. *ARID1A* variations or expressions were determined by various methods, including targeted sequencing study, immunohistochemistry (IHC), tissue microarrays, western-blot, quantitative real-time reverse transcription-polymerase chain reaction, and chromatin immunoprecipitation. The anti-*ARID1A* antibodies were inconsistent among different studies, including different antigenic determinant (monoclonal or polyclonal), various antibody manufacturer (i.e., Santa Cruz, Sigma, Abcam, and Cell Signaling Technology), diverse dilution rate (ranged from 1:1,000 to 1:200), and the different cut off that used for defining “positive *vs.* negative” samples. As shown in **Figure 2**, the frequency of *ARID1A* variations among the 29 studies ranged from 5 to 68.2%. Only one study reported that *ARID1A* variants might serve as a cancer-promoting gene in CCA, while the remaining 28 included studies indicated *ARID1A* variants might be a suppressor in CCA development and progression. Four studies have provided the data of the type of *ARID1A* variants in CCA. There are two types of *ARID1A* variants, including mutant and wild type (not mutated, the opposite of mutant type), while a mutant one is defined as loss of *ARID1A* expression or low expression of *ARID1A*. In Namjan et al.’s study (37), the authors reported that reduction of *ARID1A* expression and/or somatic mutation was associated with CCA progression. They also found that the truncation mutations (92%) of *ARID1A* were significantly associated with loss of *ARID1A* expression in CCA. In a cohort of 209 CCA cases (19), Chan-on et al. suggested the tumor-suppressive functions for *ARID1A* in CCA pathogenesis and further identified a total of 35 non-synonymous *ARID1A* somatic mutations, including 17 indels, 14 non-sense mutations, 3 missense mutations, and 1 splice-site mutation. Jiao et al. (20) reported that *ARID1A* variations occurred in 6 of 32 CCA patients (19%) and the types of mutations, including 3 non-sense mutations, 1 frameshift insertion-deletion, and 2 missense mutations. *ARID1A* variations in Simbolo et al.’s study (28) were found at 18.2% (6/33), the mutation types including 2 missense, 3 non-sense, and 1 frameshift. The characteristics of the 29 included studies are summarized in **Table 1**.

**Table 1:** Characteristics of the 28 included studies.

Author and country	Publication year	Cancer type	*ARID1A* variations (%)	Expression of *ARID1A*	Effect of *ARID1A* on CCA	Clinical Implications or biological functioning of *ARID1A*	Antibodies of *ARID1A*	Reference
Chan-on, Singapore	2013	CCA	35/209, 16.7%	Downregulated	Suppressor	*ARID1A* establishes the role of chromatin modulators in CCA pathogenesis; Silencing of *ARID1A* increases the CCA cells proliferation, while overexpression of *ARID1A* leads to retard cell proliferation	Monoclonal, Santa Cruz	(19)
Jiao, USA	2013	ICC	6/32, 19%	Downregulated	Suppressor	NA	NA	(20)
Churi, USA	2014	ICC and ECC	ICC:	Downregulated	Suppressor	*ARID1A* aberrations were not significantly associated PFS and OS in both ICC and ECC (P > 0.05)	NA	(21)
			11/55, 20%;					
			ECC:					
			1/20, 5%					
Ross, USA	2014	ICC	10/28, 36%	Downregulated	Suppressor	NA	NA	(22)
Simbolo, Italy	2014	ICC and ECC	ICC:	Downregulated	Suppressor	NA	NA	(23)
			8/70, 11.4%;					
			ECC:					
			7/57, 12.3%					
Zou, China	2014	ICC	7/102, 6.9%	Downregulated	Suppressor	NA	NA	(24)
Javle, USA	2016	ICC and ECC	ICC:	Downregulated	Suppressor	*ARID1A* variations were not significantly associated with OS in both ICC and ECC (P > 0.05)	NA	(25)
			74/412, 18%;					
			ECC:					
			7/57, 12%					
Ruzzenente, Italy	2016	ICC	4/35, 11.4%	Downregulated	Suppressor	*ARID1A* variations were independent factors related to OS (OR = 5.34, 95% CI = 1.325–21.489, P = 0.018)	NA	(26)
Sasaki, Japan	2016	ICC and ECC	ICC:	Loss of expression	Suppressor	There was no significant difference between groups with and without loss of *ARID1A* expression in OS (P = 0.9809)	Rabbit polyclonal, Sigma, 1:300	(16)
			9/49, 18.4%;					
			ECC:					
			11/72, 15.3%					
Yang, China	2016	ICC	19/57, 26.3%	Low expression	Suppressor	Low *ARID1A* expression was significantly associated with worse OS (HR = 3.97, 95% CI: 1.299–12.118, P = 0.016) in ICC; *ARID1A* was also associated with tumor nodules, vein invasion, and tumor recurrence status	Abcam, 1:1,000	(17)
Lowery, USA	2018	ICC	NA, 23%	Downregulated	Suppressor	*ARID1A* variations did not associate with metastatic site of ICC	NA	(27)
Simbolo, Italy	2018	ICC	6/33, 18.2%	Downregulated	Suppressor	*ARID1A* is an independent predictor of poor prognosis in ICC (OR = 6.9, 95% CI: 2.3–21.0, P = 0.0007)	Abcam, dilution: 1:1,000	(28)
Balitzer, USA	2019	ICC	1/7, 15%	Downregulated	Suppressor	NA	NA	(29)
Bi, China	2019	ICC	77/113, 68.2%	High expression, upregulated	Promotor	High *ARID1A* expression was correlated with the risk of death (HR = 1.95, 95% CI = 1.09–3.47) and disease recurrence (HR=2.08, 95% CI = 1.23–3.51); *ARID1A* promotes tumor initiation *via* CYP450-mediated oxidative damage	Santa-Cruz, 1:200	(18)
Joseph, USA	2019	ICC	1/10, 10%	Downregulated	Suppressor	NA	NA	(30)
Xue, China	2019	ECC	16/80, 20%	Downregulated	Suppressor	NA	NA	(31)
Conci, Italy	2020	ICC and PCC	ICC:	Downregulated	Suppressor	*ARID1A* variation is significantly associated with RFS of CCA (HR = 2.57, 95% CI = 1.174–5.608, P = 0.018); *ARID1A* variation was correlated to local recurrence of CCA	NA	(32)
			4/35, 11.4%;					
			PCC:					
			8/56, 14.3%					
Høgdall, Denmark	2020	ICC and ECC	ICC:	Downregulated	Suppressor	NA	NA	(33)
			3/13, 23%;					
			ECC:					
			6/11, 55%					
Jiang, China	2020	CCA	10/63, 15%	Downregulated	Suppressor	NA	NA	(34)
Ma, China	2020	ICC	28/130, 21.5%	Downregulated	Suppressor	*ARID1A* expressions were not significantly associated DFS and OS in patients with ICC (P > 0.05)	Abcam, 1:500	(35)
Montal, Spain	2020	ECC	21/150, 14%	Downregulated	Suppressor	NA	NA	(36)
Namjan, Thailand	2020	CCA	54/98, 55%	Loss or low expression	Suppressor	Loss or low expression of *ARID1A* exhibited distant metastasis;	Rabbit polyclonal, Sigma, 1:250	(37)
						*ARID1A* variations associated with staging, liver fluke-related status (P < 0.05)		
Sasaki, Japan	2020	Small duct ICC	13/28, 46.4%	Downregulated	Suppressor	Alteration of *ARID1A* may be involved in the carcinogenesis of small duct CCA	Rabbit polyclonal, Sigma, 1:300	(38)
Tian, China	2020	CCA	13/66, 19.7%	Downregulated	Suppressor	*ARID1A* variations were more common in ECC than ICC (31.82 *vs.* 13.64%)	NA	(39)
Wang, China	2020	ICC	20/122, 17%	Downregulated	Suppressor	NA	NA	(40)
Xu, China	2020	ICC	41/202, 20.3%	Downregulated	Suppressor	Variations frequency of *ARID1A* did not show significantly different between primary tumor and metastasis tumor samples	NA	(41)
Yoshino, Japan	2020	ICC and ECC	ICC:	Loss of expression	Suppressor	A significant correlation between *ARID1A*-negative expression and OS in ICC (HR = 2.53, 95% CI = 1.14–5.63, P = 0.023); *ARID1A* alteration induce up-regulation of multiple genes in CCA cells	Cell Signaling Technology, 1:200	(42)
			15/69, 21.7%;					
			ECC:					
			4/34, 11.8%					
Zhang, China	2021	ICC	41/318, 12.9%	Downregulated	Suppressor	NA	NA	(43)
Wagner, Germany	2021	CCA	9/52, 17%	Downregulated	Suppressor	*ARID1A* protein loss correlated with lower OS significantly (P = 0.025). Median OS of the CCA patient cohort with intact expression pattern was 39.4 ± 5.626 months *vs.* 16.125 ± 4.725 months of *ARID1A* altered patients	Abcam, 1:1,000	(44)

ARID1A, AT-rich interactive domain 1A; CI, Confidence interval; DFS, Disease-free survival; ECC, Extrahepatic cholangiocarcinoma; HR, Hazard ratio; ICC, Intrahepatic cholangiocarcinoma; NA, Not available; OR, Odds ratio; OS, Overall survival; PCC, Perihilar cholangiocarcinoma; PFS, Progression-free survival; RFS, Recurrence-free survival.

Since the variation rates, clinical implications, and the biological functioning of *ARID1A* are different among the 29 eligible studies, we perform an in-depth review on these studies as follows.

## *ARID1A* Expressions and Variations in CCA

### The Variation Rate of *ARID1A* in CCA

Comprehensive genome analysis is a useful tool for identifying various oncogene variations, especially in those genes encoding the chromatin remodeling factors. *ARID1A* is a part of the *SWI/SNF* chromatin-remodeling complex. *SWI/SNF* complexes are the most frequently mutated epigenetic regulators, controlling gene expression and cellular differentiation (45). Mounting evidence suggests *ARID1A* variation is commonly associated with multiple cancers, including gynecologic cancers, urothelial carcinoma, and gastrointestinal cancer (46, 47), with a mutational rate from 3.6 to 45.2% depending on different tumor types (48). In recent decades, more and more studies (49, 50) have found that there is a close relationship between *ARID1A* variant and the clinicopathologic features of CCA. It was reported that *ARID1A* gene variations were detected in 7.2–36% of ICC and 5–12.3% of ECC (16). However, no related review article has published focusing on this issue.

In this comprehensive review, we have summarized all the evidence relating to the *ARID1A* variation in CCA. Based on data from the 29 included studies, the variation rate of *ARID1A* varies greatly, ranging from 5 to 68.2%. As displayed in **Figure 2**, the variation rate of *ARID1A* in CCA (without distinguishing cancer type), ICC, and ECC was 15–55%, 6.9–68.2%, and 5–55%, respectively. According to previous reports, the highest *ARID1A* variant rate was found in ovarian clear cell carcinoma, up to 46–57% (42). And the *ARID1A* variation ratio was identified in about 10–20% of hepatobiliary and pancreatic cancers. Referred to the present systematic review, the variation rate of *ARID1A* was up to over 50% in some studies regardless of any type of CCA. Tian et al. (39) compared the ratio of *ARID1A* variations in the different types of CCA and found that *ARID1A* variations were more common in ECC than ICC (31.82 *vs.* 13.64%). The above results indicate that there might be a pivotal pathogenic link between the high frequency of *ARID1A* variations and CCA development.

The differences in *ARID1A* mutation rates and expression level that observed between different studies could be due to multiple factors, including distinct demographic characteristics (sample size, race, and regions), different types (ICC, ECC, or combined) and disease states (early or advanced CCA), specific anti-*ARID1A* antibodies and measurements for assessing the *ARID1A* expression (immunohistochemistry, targeted sequencing analysis, tissue microarrays, western blot, quantitative real-time reverse transcription PCR, and chromatin immunoprecipitation), and various co-present or targeted proteins being affected by *ARID1A*. Based on this evidence, an international multicenter with a large sample size and well-designed study is still needed to better illuminate the relationship between *ARID1A* mutations and CCA development, which is crucial for future strategies of CCA treatment.

Of note, except for one study reporting the significantly high level of *ARID1A* in CCA tissues, the remaining 28 studies (96.6%) suggested that the expression of *ARID1A* in CCA was low or absent. In other words, only one study concluded that *ARID1A* plays an oncogenic role, while the remaining studies suggest that *ARID1A* serves as a tumor suppressor and this is consistent with the *ARID1A* functions in other cancer types, including ovarian clear cell, gastric, pancreatic, colon, breast, lung, bladder, and renal cancer (51).

Based on the current evidence, *ARID1A* variation is expected to solely play a tumor suppressor in the tumorigenesis of multiple cancers, including CCA. Intriguingly, however, a previous retrospective study conducted by Bi et al. showed that the *ARID1A* variant might also serve as a cancer promotor in CCA development (18). They found that *ARID1A* was highly expressed in ICC tumor tissues, showing a total of 68% (77/113) tumor tissues presented with positive immunohistochemical staining in ICC. However, the majority of the clinical studies showed that decreased/absent *ARID1A* correlated with worse stage and prognosis in patients with ICC (17). This opposite effect of *ARID1A* is also detected in liver cancer. Sun et al. demonstrated that *ARID1A* had context-dependent tumor-suppressive and oncogenic roles in the liver cancers (52). The author showed that *ARID1A* was required for initial tumor development and to be inhibitory of hepatocellular carcinoma metastatic potential, indicating the role of *ARID1A* in oncogenesis was dependent on tissue context. Bi et al. (18) believed that the dual roles of *ARID1A* in CCA might be due to different sample sizes and recommended using multiple prognostic factors to avoid inconsistencies.

### Prognostic Significance of ARID1A in CCA

Though a high frequency of *ARID1A* variations is observed in the CCA, the prognostic value of *ARID1A* in CCA is still controversial. Ruzzenente et al. (26) recruited 35 patients with CCA and found that the *ARID1A* variations rate was 11.4%. The authors also found that *ARID1A* variation was an independent factor for the overall survival (OS) in CCA (OR = 5.34, 95% CI = 1.325–21.489, *P* = 0.018). The median overall survival of ICC in mutation and wild type of *ARID1A* was 14 and 52 months, respectively (*P* = 0.012). Yang et al. (17) reported that *ARID1A* variation was detected in 19/57 (26.3%) ICC patients and found that low *ARID1A* expression was dramatically correlated with worse OS (HR = 3.97, 95% CI: 1.299–12.118, *P* = 0.016). In addition, they also found that *ARID1A* was associated with tumor nodules, vein invasion, and tumor recurrence status in CCA. Conci et al. (32) indicated that *ARID1A* variation was remarkably associated with recurrence-free survival (RFS) of CCA (HR = 2.57, 95% CI = 1.174–5.608, *P* = 0.018). They further found that *ARID1A* variation was associated with a local recurrence in 43% of cases. A study (28) developed in Italy revealed that *ARID1A* variation was recorded in 18.2% and indicated that *ARID1A* variations are an independent predictor of poor prognosis in ICC (OR = 6.9, 95% CI: 2.3–21.0, *P* = 0.0007). In a study in Thailand (37), the authors found that loss or low expression of *ARID1A* was liable to distant metastasis in CCA. They also observed that *ARID1A* variations were associated with staging and liver fluke-related status (*P* < 0.05). Sasaki et al. (38) reported that the variations rate of *ARID1A* was up to 46.4% in small duct ICC and found that alteration of *ARID1A* might be involved in the carcinogenesis of CCA. Another study conducted by Sasaki et al. (52) indicated that *ARID1A* alteration was correlated with the degree of ductal plate malformation (DPM)-pattern of CCA. Yoshino et al. (42) showed a significant correlation between *ARID1A*-negative expression and OS in ICC (HR = 2.53, 95% CI = 1.14–5.63, *P* = 0.023). Wagner et al. demonstrated that suppressor *ARID1A* protein loss correlated with lower OS significantly loss of *ARID1A* protein expression is significantly correlated with lower OS when compared to the intact expression pattern (16.125 ± 4.725 *vs.* 39.4 ± 5.626 months, *P* = 0.025). The above studies that confirmed the prognostic value of *ARID1A* variations in CCA were correlated to the *ARID1A* deficiency or low expression. However, Bi et al. (18) also found that *ARID1A* alteration was associated with the risk of death (HR = 1.95, 95% CI = 1.09–3.47) as well as disease recurrence (HR = 2.08, 95% CI = 1.23–3.51), but such prognostic effects were based on high expression of *ARID1A*. Notably, an opposite effect played by *ARID1A* in CCA was found in Bi et al.’s study when compared to the remainder 28 included studies. Since Bi et al. have employed immunohistochemistry (IHC) to evaluate *ARID1A* variations rather than the sequencing analysis which was commonly used by other investigators, experimental and technical challenges (i.e. antibody clone and specificity, quality of IHC performance, etc.) might play role in the inconsistent effect of *ARID1A* identified in different studies. For example, in each of the seven studies conducted by IHC (16, 18, 28, 35, 37, 38, 42, 44), a different anti-*ARID1A* antibody is used, including the antibody’s clone, manufacturer, dilution rate, the IHC scoring, and the cut-off values. These factors could be potential reasons behind the different results obtained from each of these studies, especially the one by Bi and colleagues which is the only study that shows an oncogenic potential for *ARID1A*. The above data suggested that *ARID1A* variations regardless of low or high expression was correlated with the prognostic significance in CCA.

However, other studies have not found any correlation between *ARID1A* variation or expression level and disease prognoses in the CCA. Churi et al. (21) have identified 20 and 5% of variation for *ARID1A* in ICC and ECC, respectively. They have not found any significant correlation between *ARID1A* aberration and PFS or OS in either of ECC or ICC patients. In line with these findings, Javle et al. (25) have also found that *ARID1A* variations were not significantly associated with OS in both ICC and ECC patients (*P* > 0.05). Lowery et al. (27) demonstrated that *ARID1A* variations did not correlate with the metastatic site of ICC despite a high variant rate of 23%. Three studies developed in Asia also did not support a positive relationship between *ARID1A* variation and CCA prognosis. A study (16) in Japan revealed that there was no significant difference between groups with and without loss of *ARID1A* expression and OS in CCA patients (*P* = 0.9809). Similarly, Ma et al. (35) also demonstrated that *ARID1A* expressions were not significantly associated with DFS and OS in patients with ICC (*P* > 0.05). Xu et al. (41) reported the *ARID1A* variations rate was up to 20.3%, but the variant frequency of *ARID1A* did not show a significant difference between primary tumor and metastasis tumor samples. Based on these results, a significant correlation was not achieved between the *ARID1A* variations and the survival as well as the metastasis of CCA in these studies. A possible explanation for this phenomenon may be due to the low rate of *ARID1A* variations in CCA in some studies. For example, the *ARID1A* variations rate in ECC of Churi et al.’s study (21) was reported at only 5%, and the authors found that *ARID1A* variations were not significantly associated with PFS and OS in ECC (all P > 0.05). On the contrary, those studies reported the *ARID1A* variations rate over 20% were more likely to more likely to identify a poor prognostic significance for *ARID1A* variations in CCA (17, 18, 37, 42).

Collectively, *ARID1A* variations might be an important prognostic indicator that can predict disease mortality, metastasis, and recurrence in CCA patients, which also suggests that *ARID1A* could play important roles in the CCA progression and worth more attentions. Of note, since several studies have not supported such a prognostic value for *ARID1A* further studies with larger sample sizes are warranted to validate the prognostic values of *ARID1A* variations in CCA.

### Biological Functions of ARID1A and Its Pathological Impact on CCA

Since a causal relationship between *ARID1A* alteration and CCA is suggestive from many clinical studies, a better understanding of the biological functions of *ARID1A* and its underlying mechanisms in CCA development is profound for the investigators. *ARID1A* is a driver gene encoding the DNA-binding subunit of the *SWI/SNF* chromatin-remodeling complexes. *ARID1A* provides specificity for *SWI/SNF* complex, facilitating protein­protein or protein­DNA molecule interactions. Knockdown of *ARID1A* gene could induce dysregulation of cell cycle arrest thus enhance tumorigenesis (53). Inactivation of *ARID1A* might activate cell cycle progression, leading to an uncontrolled cellular proliferation in cancer cells. *ARID1A* commonly exerts the tumor-suppressive functions in CCA as well as multiple cancers. It was suggested that frequent variations in *ARID1A* related to perturbation in chromatin remodeling and chromosome organization might participate in the carcinogenesis and progression in CCA (54). Chan-on et al. (19) reported that *ARID1A* played a role of chromatin modulator in CCA pathogenesis, showing that silencing of *ARID1A* enhanced the CCA cells proliferation and upregulation of *ARID1A* causes disruption of cell proliferation. Yoshino et al. (42) indicated that *ARID1A* alteration could induce up-regulation of multiple genes (i.e., *ALDH1A1*, Aldehyde dehydrogenase 1A1, a potent cancer stem cell marker) in CCA cells. *ARID1A*-knockout in CCA cells lines promotes migration, invasion, and sphere formation activity, which might be correlated to transcriptional suppression of *ALDH1A1* expression with decreasing histone H3K27 acetylation. A study conducted by Sasaki et al. (16) suggests that loss of *ARID1A* expression might be an early event in CCA development which presents a novel molecular pathway that is characterized by non-papillary and tubular adenocarcinoma. A precursor lesion with loss of *ARID1A* expression might cause a premalignant lesion of cholangiocarcinoma. *TP53*, also known as *P53*, is one of the most frequent genetic variants in human cancers, which plays a key role in the control of the cell cycle, apoptosis, and DNA repair (55). Alteration of *PT53* is a prognostic biomarker for cancer due to its biological function of carcinogenesis. A meta-analysis indicated that *TP53* might be a pivotal prognostic factor for the OS of patients with ECC (56). Both *TP53* and *ARID1A* are frequently mutated in patients with CCA for their chromatin remodeling function. Interestingly, many researchers have found that *TP53* and *ARID1A* variations appeared simultaneously in CCA (57). It was reported that *ARID1A* and *P53* collaborated to prevent tumorigenesis by transcriptional activation of the tumor-inhibiting downstream genes (48). Therefore, the prognostic value and the molecular biological effect of *ARID1A* in CCA might partially depend on the alteration of *TP53*. Sasaki et al. (58) developed a study related to the combined hepatocellular-cholangiocarcinoma (cHC-CC) and found that the effect of *ARID1A* variations on the clinicopathological significance of cHC-CC might be correlated to oxidative stress and alpha-fetoprotein (AFP)-positivity. Farshidfar et al. (59) showed that *ARID1A* exhibited DNA hypermethylation and decreased expression of the isocitrate dehydrogenase (IDH) in the mutant subtype of CCA.

In a more recent study (60) recruited of 412 intrahepatic CCA, Boerner et al. found that both *ARID1A* (20%) and *TP53* (17%) were among the most common oncogenic alterations in CCA. In line with this finding, Zhang et al. (43) also reported that both *TP53* and *ARID1A* were among the most frequently mutated genes in intrahepatic CCA. In addition to *TP53*, *ARID1A* variations can co-occur and probably interact with multiple other genes (i.e., *ALDH1A1*, *Beclin-1*, *BAP1*, and *PBRM1*) which could be involved in CCA development. For example, Jiao et al. (20) demonstrated that genes involved in chromatin remodeling (including *BAP1*, *ARID1A*, and *PBRM1*), which was considered as the frequently targeted pathway in CCA, were somatically altered in almost half of the intrahepatic CCA cases. In 2013, Chan-on et al. (19) identified *ARID1A* and *BAP1* as two new genes mutated in CCA. They further found that these two genes showed typical features of tumor suppressors and their mutations were mainly truncating and were scattered throughout the entire gene. However, we should also note that *ARID1A* might play an independent role in CCA development. Because some other studies [i.e. Sasaki et al. (16)] have failed to find CCA harboring both *ARID1A* and *KRAS* mutations (another frequently mutated gene in CCA), whereas these tumors had lost *ARID1A* expression suggesting that loss of *ARID1A* expression might represent an alternative (to *ARID1A* genomic variations) mechanism for *ARID1A*-driven carcinogenesis in CCA, and this could also be an alternative to *KRAS* mutations-driven CCA development. In line with this, Namjan et al. (37) have not been able to identify the co-occurrence of *ARID1A* and *TP53/KRAS* mutations in some CCA cases.

**Figure 3:**
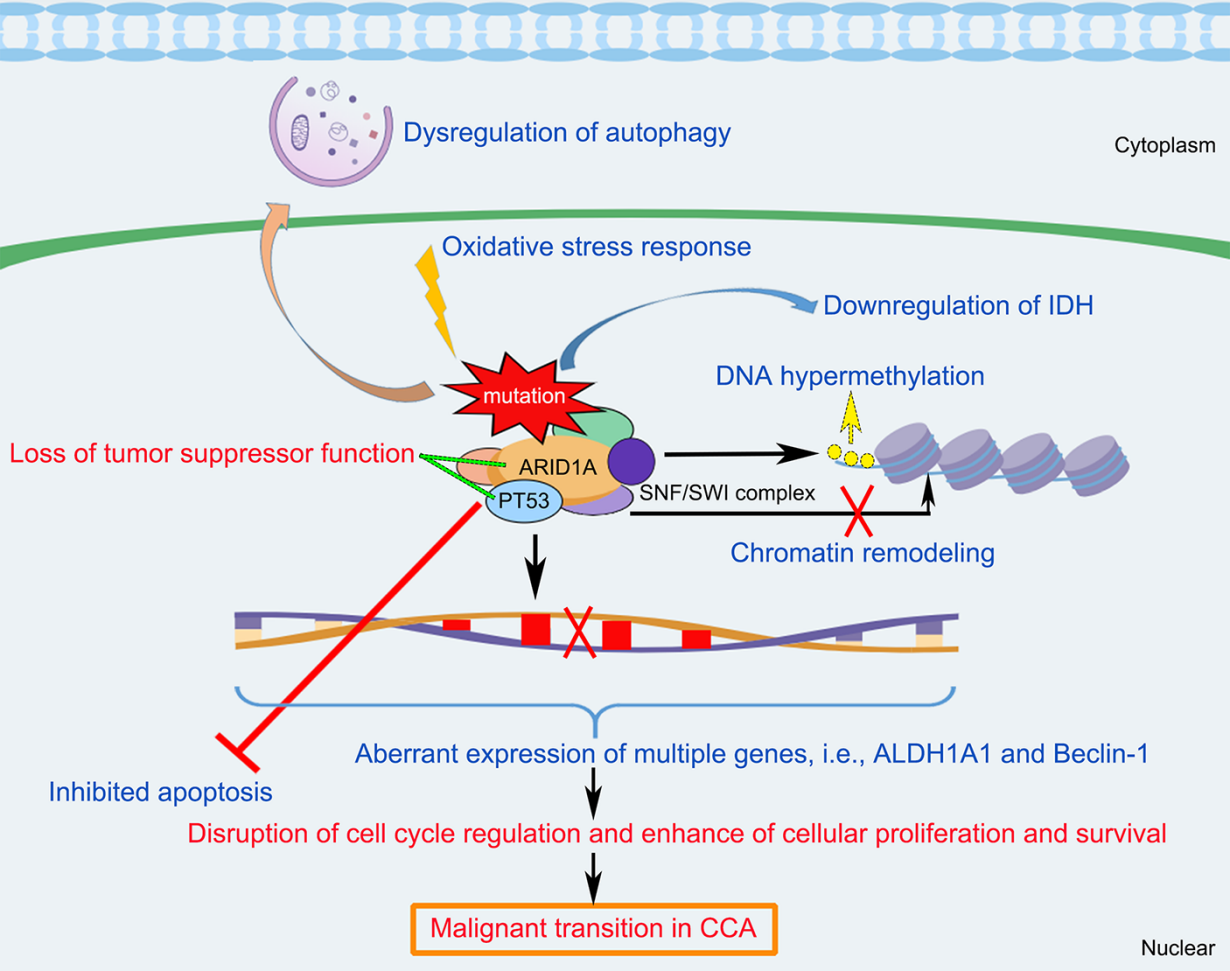
Schematic diagram of the molecular mechanisms underlying *ARID1A* variant and the pathogenesis and pathophysiology of CCA. *ARID1A* alteration associated with disruption of cell cycle regulation, chromatin remodeling, oxidative stress damage, DNA hypermethylation, downregulation of IDH, and the interaction of multiple genes (i.e., *PT53*, *ALDH1A1*, and *Beclin-1*), resulting in inhibition of apoptosis, dysregulation of autophagy, and enhance of cellular proliferation and survival, which cause loss of tumor suppressor functioning of *ARID1A* and induce malignant transition in CCA. *ALDH1A1*, Aldehyde dehydrogenase 1A1; *ARID1A*, AT-rich interaction domain 1A; IDH, isocitrate dehydrogenase; *SWI/SNF*, SWItch/Sucrose Non-Fermenting.

In summary, *ARID1A* variations appear to play important roles in the CCA tumorigenesis and progression. As shown in **Figure 3**, this schematic diagram summarizes that multifactorial mechanisms that are potentially involved in the *ARID1A*-driven CCA development, including opposing functions in cell cycle arrest, chromatin remodeling and chromosome organization, oxidative stress damage, DNA hypermethylation, downregulation of IDH, and the interaction of multiple genes (i.e., *TP53*, *ALDH1A1*, and *Beclin-1* target) that enhance cellular proliferation and anti-apoptotic processes. However, further comprehensive researches are still warranted to better elucidate the underlying mechanisms of CCA development initiated by *ARID1A* variations.

### Limitations and Perspectives

To our knowledge, this is the first study for conducting a comprehensive review to summarize all the evidence of the relationship between *ARID1A* variations and CCA at both clinical and biological levels. However, some inherent limitations should be noted. First, the variation rate of *ARID1A* in CCA diverse in different studies, ranging from 5 to 68.2%. Different cancer types and stages, study design, sample size, geographical areas, gender, and age could all be partly responsible for this heterogeneity. Second, the prognostic significance of *ARID1A* in CCA is still controversial among the included studies. Nevertheless, we do not perform a meta-analysis for these studies due to due to unavailability of sufficient relevant data (i.e., OS, PFS, RFS, DFS, and metastatic site) in most of the studies. Third, the molecular mechanisms for the involvement of *ARID1A* variants in CCA tumorigenesis remain to be explored. In addition to *ARID1A* alteration, some other common contributors in cancer development, such as tumor microenvironment and tumor immunity, might also play roles in the pathomechanism of CCA, which should be further investigated.

## Conclusions

This review has shown *ARID1A* variations in CCA cases are diverse in different studies, ranging from 5 to 68.2%. A higher *ARID1A* variation rate has been identified in the ICC subgroup when compared to the ECC subgroup (6.9–68.2% *vs.* 5–55%). Excepting for one study indicating the carcinogenic functions for *ARID1A*, the remaining 28 included studies suggest that *ARID1A* is a tumor suppressor in CCA. Though more studies show that loss or low *ARID1A* expression is significantly correlated with worse survival and recurrence in CCA, several studies do not support these prognostic values of *ARID1A*. Multifactorial mechanisms are involved in the pathogenesis of CCA caused by *ARID1A* variations. More investigations are necessary to validate the prognostic significance of *ARID1A* variations and to identify its molecular mechanism in CCA development.

## Author Contributions

SZ, YX, and WW contributed to conceive and design the study. YW and PW performed the systematic searching. HJ and JZ extracted the data. SZ and JZ wrote the manuscript. YX and WW supervised the manuscript. All authors contributed to the article and approved the submitted version.

## Funding

This work was supported by the Welfare Technology Applied Research Project of Zhejiang Province (LGC20H200004), Reagent Project of Taizhou City, Zhejiang Province (ID: 1901ky38), the High-level Hospital Construction Research Project of Maoming People’s Hospital, and the Technology Planning Project of Taizhou City, Zhejiang Province (ID: 20ywb40).

## Conflict of Interest

The authors declare that the research was conducted in the absence of any commercial or financial relationships that could be construed as a potential conflict of interest.
